# Tunable Resonators for Nonlinear Modal Interactions

**DOI:** 10.1038/srep34717

**Published:** 2016-10-04

**Authors:** Abdallah H. Ramini, Amal Z. Hajjaj, Mohammad I. Younis

**Affiliations:** 1 Physical Sciences and Engineering Division, King Abdullah University of Science and Technology Thuwal 23955-6900, Saudi Arabia; 2 Department of Mechanical Engineering, State University of New York at Binghamton, Binghamton, NY 13902, USA.

## Abstract

Understanding the various mechanisms of nonlinear mode coupling in micro and nano resonators has become an imminent necessity for their successful implementation in practical applications. However, consistent, repeatable, and flexible experimental procedures to produce nonlinear mode coupling are lacking, and hence research into well-controlled experimental conditions is crucial. Here, we demonstrate well-controlled and repeatable experiments to study nonlinear mode coupling among micro and nano beam resonators. Such experimental approach can be applied to other micro and nano structures to help study their nonlinear interactions and exploit them for higher sensitive and less noisy responses. Using electrothermal tuning and electrostatic excitation, we demonstrate three different kinds of nonlinear interactions among the first and third bending modes of vibrations of slightly curved beams (arches): two-one internal resonance, three-one internal resonance, and mode veering (near crossing). The experimental procedure is repeatable, highly flexible, do not require special or precise fabrication, and is conducted in air and at room temperature. This approach can be applied to other micro and nano structures, which come naturally curved due to fabrication imperfections, such as CNTs, and hence lays the foundation to deeply investigate the nonlinear mode coupling in these structures in a consistent way.

Nonlinear mode coupling among the vibration modes has been reported numerously in micro and nano structures in recent years^1^,^2^,^3^,^4^,^5^,^6^,^7^,^8^,^9^. It represents a mechanism for energy leakage from the intentionally excited mode of vibration, which typically is targeted for detection and measurements, to other unintentional modes of vibrations, which often are not monitored or detected. Therefore, if activated, it can represent a significant source of energy loss. Hence, it might be responsible to some extent for the low quality factor reported in nano structures, such as CNTs and Graphene membranes, even at very low vacuum.

On the other hand, energy transfer through nonlinear mode coupling has been proposed for useful potential applications, such as energy harvesting and mass sensing^9^,^10^,^11^,^12^,^13^,^14^,^15^,^16^,^17^. More important, recently, it has been proposed as a mechanism to suppress noise^7^ and stabilize oscillation in MEMS resonators for frequency referencing^18^,^19^,^20^,^21^,^22^,^23^.

There are at least two major mechanisms of mode coupling that have been reported. The first is internal resonance among the various modes of vibrations. These can be all in the same plane^24^,^25^,^26^ or of different planes^7^,^8^,^9^,^26^. The ratio of the various resonance modes involved in the coupling can be one-one^9^,^24^,^26^, two-one^8^,^25^,^26^,^27^, and three-one^7^,^25^,^26^,^27^. The second mechanism is through mode veering, also called near crossing. In this mechanism, the natural frequencies of two modes get relatively close to each other and then deviate, veer, away after some parameter change. This mechanism particularly has been reported in slightly curved, slacked, CNTs, which shows potential transfer of energy from the first targeted mode to up to the ninth (untargeted) mode^1^,^28^.

Despite the recent progress in characterizing and testing nonlinear mode coupling at the micro and nano scale^7^,^8^, establishing well-controlled, repeatable, and flexible experimental procedure to probe this phenomenon remains challenging. There are no clear guidelines to help understand how to tune the resonance frequencies of a structure to have certain ratios (one-one, two-one, three-one, or near each other in the case of veering). This is particularly challenging due to the unavoidable imperfections that arise during the fabrication process, which makes adjusting exact ratios to realize such nonlinear interactions very challenging. To advance knowledge in identifying and studying nonlinear mode coupling at the micro and nano scale, structures of highly tunable resonance frequencies are desired. At the same time; this tunability should not be highly dependent on fabrication accuracy.

We present in this study an experimental approach that resolves the aforementioned issues. Particularly, we demonstrate using electrothermal tuning and electrostatic actuation to excite slightly curved structures, shallow arches, into various types of internal resonances, particularly, two-one, three-one, and veering. Such curved structures are very common. At the micro scale, they show up in out-of-plane fixed-fixed structures due to stress gradient and the deposition of layers of different thermal expansion coefficients. At the nano scale, they appear in the form of slack in CNTs and graphene sheets. Here, for the purpose of generating a controlled experiment, we intentionally fabricate in-plane silicon resonators of predetermined curvature. Even if the deliberate curvature does not come out exactly after fabrication as intended, as will be demonstrated, thermal tuning can adjust this curvature as needed.

## Results

### Devices

**Figure 1:**
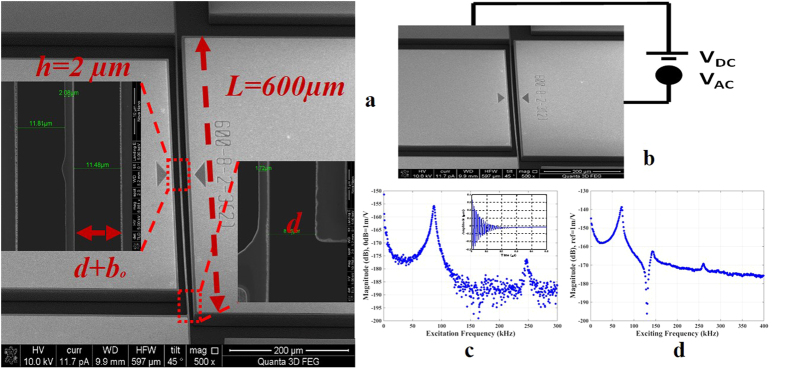
(**a**) An SEM image showing the top view of an in-plane shallow arch and its dimensions. The arch beam is sandwiched between two silicon pads to maintain isotropy during Deep Reactive Ion Etch (DRIE) layer etching and the removal of the oxide layer underneath the vibrating beam. (**b**) An SEM image of the arch with a schematic showing the electrostatic actuation (V_DC_ and V_AC_) connection. (**c**) Frequency spectrum measurement of arch A showing the 1^st^ and 3^rd^ resonance frequencies. The arch is separated from the lower actuating electrode by a gap of 8.86 μm near the anchors. It has an initial curvature of 2.86 μm at its mid-point. The 1^st^ resonance frequency of arch A is found at 86.6 kHz corresponding to the first symmetric mode shape and its quality factor Q = 19.74. Its 3^rd^ resonance frequency is 247 kHz with a quality factor of 4.5. The inset shows the time history of the ring down measurement. (**d**) Frequency spectrum measurement for arch B showing the 1^st^ resonance frequency at 64 kHz with a quality factor of 19.74 and the 3^rd^ resonance frequency at 251 kHz with a quality factor of 5.31. The arch is separated from the lower electrode with a gap of 7.4 μm and it has an initial curvature at the mid-point of 2.5 μm.

We study in-plane MEMS shallow arch resonators fabricated using a highly conductive Si device layer of SOI wafer of thickness 25 μm by a two-mask fabrication process by MEMSCAP. Each resonator is clamped from both ends and is actuated electrostatically using a stationary electrode. Also, it is actuated electrothermally by passing a DC current through its ends, which heats it up through Joule’s heating and induces an axial stress inside it. The dimensions of the studied arches, Fig. 1a, are 600 μm in length, 25 μm in width, and 2 μm in thickness.

### Resonance Frequencies at Zero Thermal Load

Two case studies of arches are considered: arch A and arch B. Both share same geometrical properties, but differ in the internal axial stress induced during microfabrication and in their initial curvature. First, we stimulate the two arches electrostatically (Fig. 1b) by using a ring down technique (Fig. 1c,d) to reveal their first and third resonance frequencies. For arch B, it was difficult to reveal the 3^rd^ resonance frequency by using the ring down measurement because the response was within the noise level. For this, we used a frequency sweep test to obtain the 3^rd^ resonance frequency while maintaining the linear behavior by using a small electrostatic voltage.

### Electrothermal Frequency Shifting

**Figure 2:**
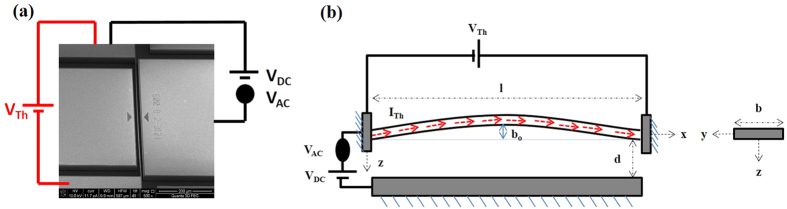
(**a**) An SEM image of an arch showing schematically electrostatic and electrothermal actuation. (**b**) A schematic shows an arch actuated electrothermally via V_Th_ through its ends and electrostatically via V_DC_ and V_AC_. The arrows in red color represent a current passing through the arch beam, which induces axial stress and tunes its resonance frequencies.

Next, we use electrothermal actuation, Fig. 2, by passing an electrical current generated by a constant DC voltage V_Th_ to induce axial stresses; and hence tune the resonance frequencies of the arches.

**Figure 3:**
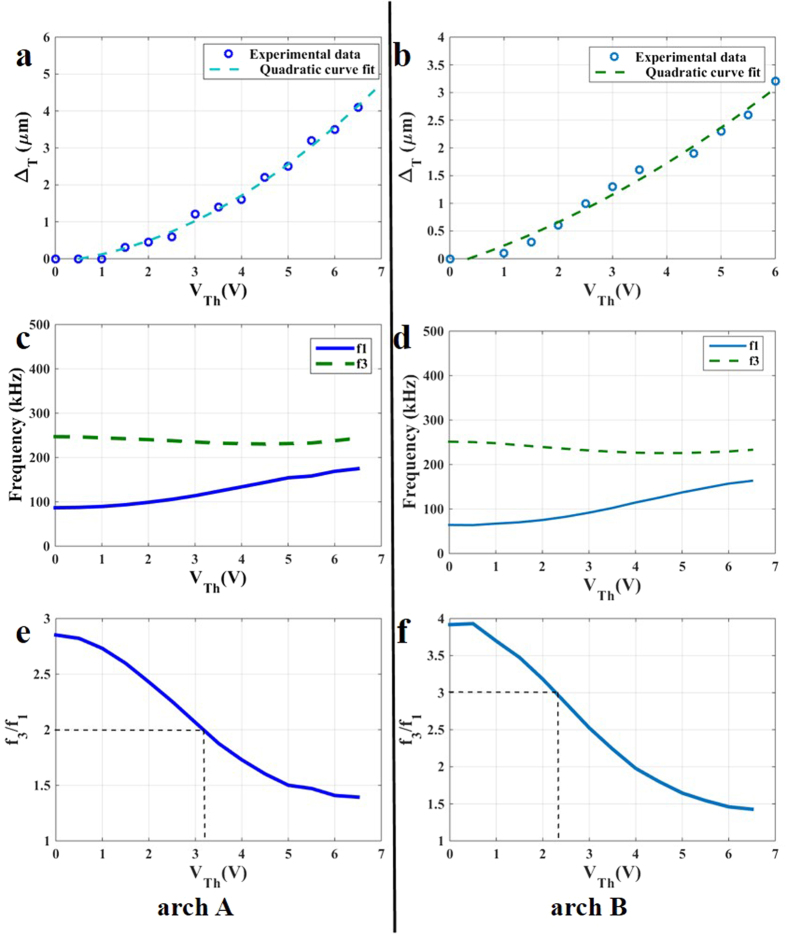
Electrothermal tuning of the arches. (**a**,**c**,**e**) are for arch A, (**b**,**d**,**f**) for arch B. (**a**) The deflection of the middle of arch A, Δ_T_, versus V_Th_ showing the increase in its curvature. (**b**) The deflection of the middle of arch B, Δ_T_, versus V_Th_. (**c**) The shift in the resonance frequencies of arch A from their initial values under the effect of V_Th_: the 1^st^ resonance frequency f_1_ increases and the 3^rd^ resonance frequency f_3_ decreases. (**d**) The shift in the resonance frequencies of arch B under the effect of V_Th_. (**e**) The ratio between the 1^st^ and 3^rd^ resonance frequencies decreases when V_Th_ is increased for arch A. The dashed line shows the value of V_Th_, 3.2 V, at which f_3_/f_1_ = 2. (**f**) The ratio between the 1^st^ and 3^rd^ resonance frequencies for arch B while varying V_Th_. The dashed line shows the value of V_Th_ = 2.45 V at which f_3_/f_1_ = 3.

Applying V_Th_ generates axial stresses along the arches, thereby increasing their curvature (Fig. 3a,b). This shifts their resonance frequencies where the 1^st^ resonance frequency increases while the 3^rd^ resonance frequency decreases (Fig. 3c,d). More important, the ratio between these frequencies tends to decrease until it reaches a regime where it becomes almost flat (Fig. 3e,f).

As noted from Fig. 3e,f, the two arches have different starting frequency ratios of f_3_/f_1_ = 2.85 for arch A (Fig. 3c) and =3.92 for arch B (Fig. 3f). In arch A, one type of internal resonance can be activated, which is 2:1. In arch B, on the other hand, we can have two types of internal resonance: 2:1 and 3:1. Here, we focus on the cases of f_3_/f_1_ = 2 for arch A and f_3_/f_1_ = 3 for arch B.

## Internal Resonance

Next, we experimentally demonstrate the 2:1 and 3:1 internal resonances of the arches under investigation.

### 2:1 Internal Resonance

**Figure 4:**
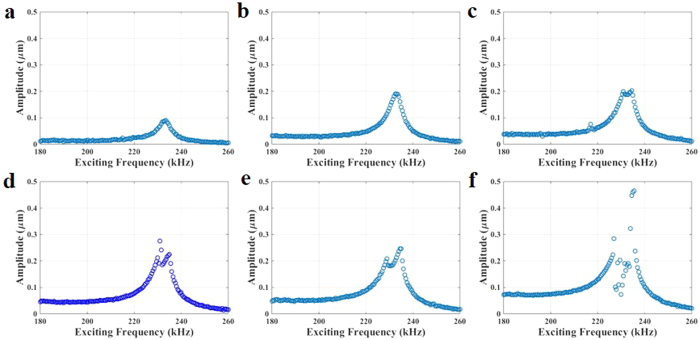
Internal resonance revealed via forward frequency sweeps in arch A at a constant V_Th_ = 3.2 V, where the frequency is swept around the 3^rd^ resonance frequency for different values of actuation voltage. The arch behaves linearly at (**a**) V_DC_ = 20 V and V_AC_ = 20 V; and (**b**) V_DC_ = 30 V and V_AC_ = 30 V. Then, the arch experiences internal resonance at voltage loads of (**c**) V_DC_ = 30 V and V_AC_ = 40 V, (**d**) V_DC_ = 30 V and V_AC_ = 50 V, (**e**) V_DC_ = 40 V and V_AC_ = 40 V, and (**f**) V_DC_ = 40 V and V_AC_ = 60 V.

To demonstrate the 2:1 internal resonance in arch A, we sweep the frequency of the electrostatic voltage around the 3^rd^ resonance frequency when V_Th_ = 3.2 V. At small electrostatic voltages, the frequency response curve around the 3^rd^ resonance frequency behaves linearly (Fig. 4a,b). As the electrostatic voltage is increased, the arch resonator starts to experience internal resonance, where the vibrational amplitude deflection splits and two peaks of vibrational amplitude emerge around the 3^rd^ resonance frequency (Fig. 4c). This splitting of the frequency response curve is a typical behavior of nonlinear mode coupling due to a 2:1 internal resonance (see Figs S1.1 and S1.2a). Increasing the electrostatic voltage, the separation between the peaks increases, Fig. 4d,e. Finally, Fig. 4f shows coexistence of states and what seems to be a Hopf bifurcation, which was reported for macro structures^26^.

A remark is worth to be noticed here is regarding the apparent broadening of the resonance band due to the presence of two peaks in the frequency response curve. Particularly, Fig. 4c shows almost flat high amplitude compared to a single amplitude of narrow band width in Fig. 4b. This may be promising for band-pass filtering applications.

**Figure 5:**
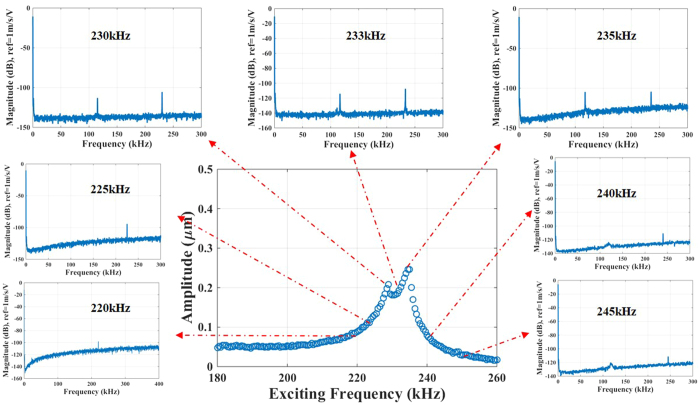
Experimental FFTs of arch A for various excitation frequencies of the frequency response curve for V_DC_ = 40 V and V_AC_ = 40 V when V_Th_ = 3.2 V. Note that the FFT shows a single spike at 220 kHz and 225 kHz corresponding to the excitation frequency with no indication of nonlinear model coupling. On the other hand, a second spike, in addition to that at the excitation frequency, emerges at 230 kHz, 233 kHz, and 235 kHz. The second spike in the FFT always is spotted at half the excitation frequency, which happens to be close to the first resonance frequency of the arch. This indicates a 2:1 internal resonance and nonlinear mode coupling among the first and third modes. The second spike diminishes in strength as passing the internal resonance regime at 240 kHz and 245 kHz.

To verify the existence of the 2:1 internal resonance in arch A, one needs to examine the Fast Fourier Transformation FFT of the response for several values of excitation frequencies near the internal resonance regime. Note here that the FFT requires processing a time history signal of the response, which is currently not possible using our in-plane measurement technique that relies on image capturing of the last period of the steady-state response. Hence, we resort instead to measuring the out-of-plane response of the arch, which relies on the fact that the in-plane motion will result in measurable motion in the out-of-plane direction. We used a Laser Doppler Vibrometer (LDV) to acquire the out-of-plane measurements. The FFT shows one peak before internal resonance at 220 kHz and 225 kHz excitation frequencies (Fig. 5). Then as passing the internal resonance regime, the FFT shows two frequencies: one at the excitation frequencies (230 kHz, 233 kHz and 235 kHz) that is close to the 3^rd^ resonance frequency and one at the 1^st^ resonance frequency, which almost equals half of the 3^rd^ resonance frequency. After passing internal resonance, the first resonance peak in the FFT measurements dissipates at 240 kHz and finally diminishes at 245 kHz. Hence, these results confirm that the 2:1 internal resonance is activated.

**Figure 6:**
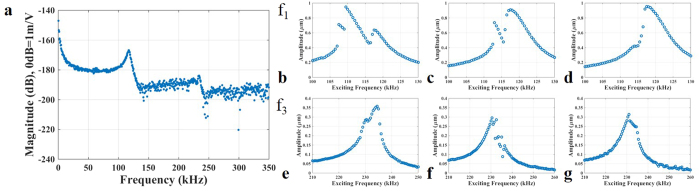
(**a**) In-plane frequency spectrum measurement of arch A at V_Th_ = 3.2 V confirming f_3_ = 2 f_1_ = 230 kHz. (**b**) While fixing the electrostatic voltage at V_DC_ = 40 V and V_AC_ = 45 V, the frequency is swept around the neighborhood of f_1_ when V_Th_ = 3.2 V. Two peaks emerge on the frequency response around f_1_. (**c**) When V_Th_ = 3.4 V, the two peaks get closer to each where the second peak gains a higher amplitude than that of the previous one. (**d**) When V_Th_ = 3.5 V, the two peaks merge to form one peak around f_1_. Also, the frequency is swept around the neighborhood of the 3^rd^ resonance frequency for the previous cases. (**e**) V_Th_ = 3.2 V, (**f**) V_Th_ = 3.4 V, (g) V_Th_ = 3.5 V.

Next, we explore the effect of varying V_Th_ on the 2:1 internal resonance. Changing V_Th_ changes the ratio of the resonance frequencies; and hence can be viewed as a detuning parameter that controls the strength of the mode coupling. First, we confirm for the chosen voltage load that the ratio of the resonance frequencies of the first and third modes is 2:1, Fig. 6a. The detuning effect is then examined near the vicinity of the first resonance frequency, Fig. 6b–d, and near the third resonance frequency, Fig. 6e–g. This reported qualitative change in the frequency response is similar to the theoretical predictions of Fig. S.2b in Supplementary Material, which shows the effect of the detuning similar to that of V_Th_.

### 3:1 Internal Resonance

**Figure 7:**
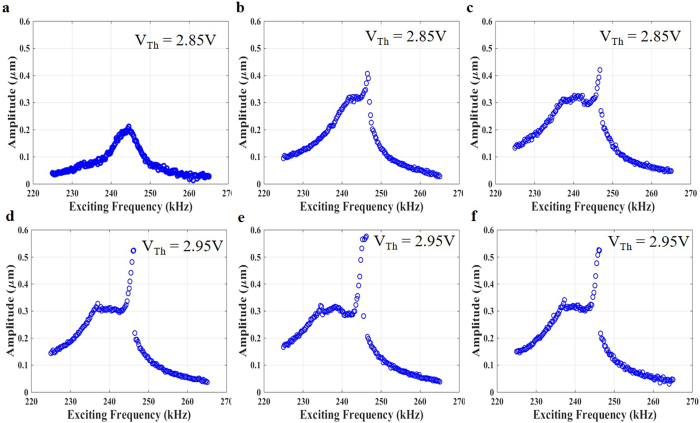
Frequency sweeps revealing a 3:1 internal resonance at V_Th_ = 2.85 V–2.95 V, where the frequency is swept around the 3^rd^ resonance frequency regime for different values of actuation voltage. The arch behaves linearly at (**a**) V_DC_ = 30 V and V_AC_ = 30 V. After that, the arch experiences internal resonance at (**b**) V_DC_ = 30 V and V_AC_ = 60 V. (**c**) V_DC_ = 30 V and V_AC_ = 80 V. (**d**) V_DC_ = 30 V and V_AC_ = 80 V. (**e**) V_DC_ = 30 V and V_AC_ = 90 V. (**f**) V_DC_ = 40 V and V_AC_ = 60 V.

One of the advantages of an arch beam is its ability to produce several types of internal resonance when changing its curvature. Here, we demonstrate a 3:1 internal resonance for arch B as an example. For this, we sweep the frequency of the electrostatic voltage around the 3^rd^ resonance frequency regime when V_Th_ = 2.85 V (Fig. 7a–c) and 2.95 V (Fig. 7d–f). At small electrostatic voltages, the frequency response curve around the 3^rd^ resonance frequency is linear (Fig. 7a). Increasing the electrostatic voltage, the arch resonator experiences an internal resonance, where a flat response near the primary resonance is observed with a distinctive peak emerging near the end of this flat regime (Fig. 7a–f). The flat amplitude in the frequency response curves is wider and flatter than that in the 2:1 internal resonance.

**Figure 8:**
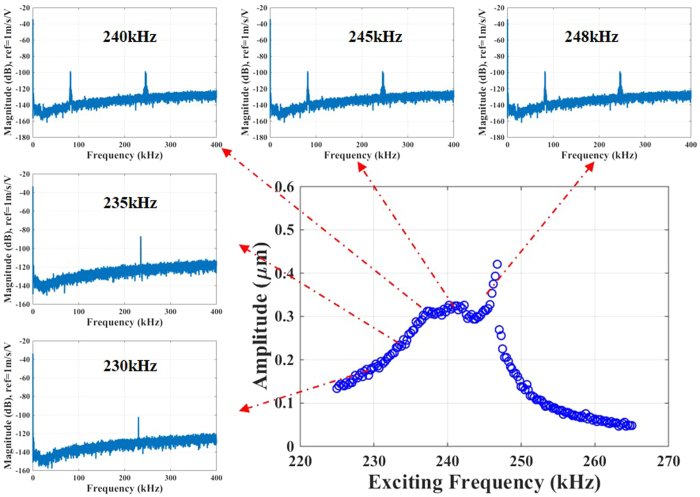
Out-of-plane FFTs measured for arch B for several excitation frequencies on the frequency response curve during a 3:1 internal resonance for the case of V_DC_ = 30 V, V_AC_ = 80 V, and V_Th_ = 2.85 V. Note the emergence of two spikes in the FFTs (one at the excitation frequency and one at the first resonance frequency) at 240 kHz, 245 kHz and 248 kHz during the internal resonance compared to a single spike at the excitation frequencies of 230 kHz and 235 kHz outside the regime. The widening of the spikes during the internal resonance can be attributed to the Hopf bifurcation^26^, which generates new frequencies that might be incommensurate with the original frequencies.

As in the previous case of the 2:1 internal resonance, we acquire the out-of-plane FFT measurements for the in-plane motion of arch B to verify the existence of internal resonance and modal interaction (Fig. 8). Before the flat amplitude regime, the FFT reveals a single peak at the excitation frequency. Within the flat amplitude, the FFT shows two peaks of frequencies at the excitation frequencies and at one third of the excitation frequencies, which is close to the first resonance frequency of the arch. (See Supplementary Material).

### Veering

Another intriguing interaction among the vibration mods of micro and nano resonators is veering^1^, also called near crossing. Modes veering offers a stronger way of modal energy transfer compared to the internal resonance phenomenon because the coupling among the modes occurs at the eigenvalue level. Hence, it does not require high level of excitation/nonlinearity to be activated. Its ingredient is to have two natural frequencies approaching each other while changing a bifurcation parameter. After veering, the two involved modes “almost” exchange roles and shapes.

**Figure 9:**
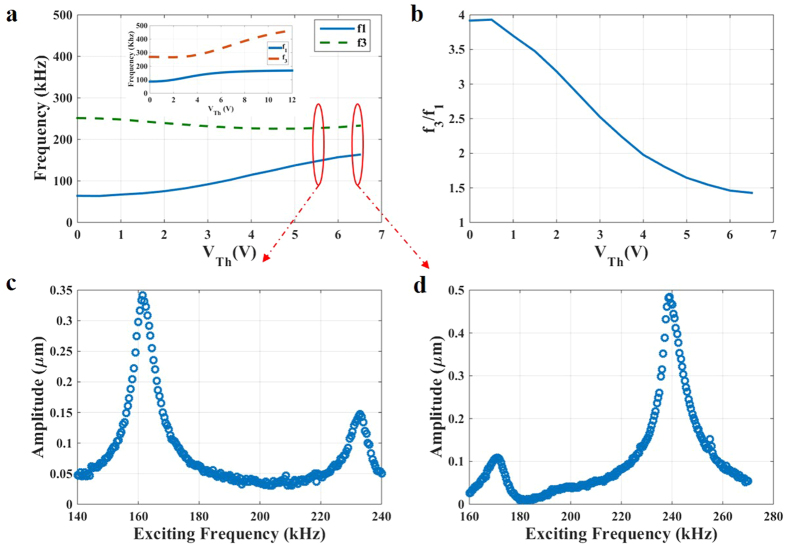
Modal interaction and veering demonstration. (**a**) Variation of the first and third resonance frequencies with V_Th_. The inset shows the numerical simulation results based on a reduced-order model (Supplementary Material). (**b**) The ratio between the first and third resonance frequencies showing a continuous decrease with V_Th_ until reaching saturation regime. (**c**) Just before veering at V_Th_ = 5.5 V, the frequency is swept from the 1^st^ resonance frequency regime to the 3^rd^ resonance frequency regime for V_DC_ = 30 V and V_AC_ = 40 V. (**d**) After veering at V_Th_ = 6.5 V, the frequency is swept from the 1^st^ resonance frequency regime to the 3^rd^ resonance frequency regime V_DC_ = 50 V and V_AC_ = 50 V. Interestingly, the coupling between the modes after veering amplifies the vibrational amplitude of the 3^rd^ resonance more than that of the 1^st^ resonance frequency.

We demonstrate here that V_Th_ can be used as a bifurcation parameter to induce veering. To demonstrate this, we consider arch B. Figure 9a reveals the variation of the first two resonance frequencies of the arch with V_Th_. The figure indicates that the resonance frequencies of the two modes approach each other and then depart away. Figure 9b highlights this by displaying the ratio among the two frequencies as varying V_Th_, which shows the ratio drops from almost 4 to 1.4. Increasing V_Th_ makes these two frequencies depart way from each other. This is clearer in the inset of Fig. 9a based on the theoretical simulations (see also Supplementary Material).

Next, we conduct frequency sweep tests for arch B using electrostatic excitation in the neighborhood of the two involved frequencies; slightly below and after the veering regime of Fig. 9a. Before veering, the 1^st^ resonance frequency on the frequency response curve has higher vibrational amplitude than that of the 3^rd^ resonance frequency (Fig. 9c). After veering, this interaction between both modes increases the vibrational amplitude of the 3^rd^ mode to surprisingly higher amplitudes than that of the 1^st^ mode (Fig. 9d). This result can be promising since it shows a method to make the higher-order modes of vibration more sensitive to axial stress variation and also to external excitations, by making them operating beyond the veering point. Hence, the resonator becomes more responsive to the excitation forces, and hence it requires less power to excite and responds at higher amplitude compared to noise.

## Discussion

We demonstrated that several interactions among the vibration modes can be activated through electrothermal modulation. We showed these through frequency sweep tests that tracked the variation in the amplitude of motion during internal resonance and veering. These mode couplings are driven by weak electrostatic forcing. Interestingly, all the demonstrated phenomena were conducted at atmospheric pressure and room temperature.

In conclusion, we have demonstrated a systematic approach to tune the ratios among the natural frequencies of micro and nano resonators through electrothermal actuation Through electrothermal and electrostatic actuation, we successfully demonstrated the activation of several nonlinear interaction phenomena; mainly 2:1 and 3:1 internal resonances as well as veering. All these have been demonstrated in the same category of structures of a MEMS arch resonator. The demonstrated procedure does not require any special fabrication. Hence, it can be used to investigate in more depth these and other similar nonlinear interaction phenomena at the micro and nano scale in a well-controlled setting. The ability to tune and modulate the resonance frequencies and their ratios opens the possibility to explore in depth the nonlinear dynamics of MEMS and NEMS oscillators and to more aggressively exploit internal resonances and modal interactions.

## Methods

### Arch Resonator Amplitude and Frequency

The motion of the resonator was detected optically using a stroboscopic video microscopy for in-plane motion analysis. After reaching steady state vibration, the amplitude of the last period was calculated at each frequency step of the frequency sweep. Then, we generated and constructed the frequency response curve for each voltage combinations. For the out-of-plane motion and the FFTs, a Laser Doppler Vibrometer is used.

## Additional Information

**How to cite this article**: Ramini, A. H. *et al.* Tunable Resonators for Nonlinear Modal Interactions. *Sci. Rep.* **6**, 34717; doi: 10.1038/srep34717 (2016).

## Supplementary Material

Supplementary Information

Supplementary Video 1
