# Supplementary material for: Tunable Resonators for Nonlinear Modal Interactions
Source: Sci Rep. 2016 Oct 4;6:34717. doi: 10.1038/srep34717 (PMC5048135; doi:10.1038/srep34717)
Supplement: Supplementary Information [file srep34717-s1.pdf]

Tunable Resonators for Nonlinear Modal Interactions

Abdallah Ramini[1], Amal Hajjaj[1] and M.I Younis[1, 2]

[1]Physical Sciences and Engineering Division, King Abdullah University of Science and Technology Thuwal 23955-6900, Saudi Arabia.

[2]Department of Mechanical Engineering, State University of New York at Binghamton, Binghamton, NY 13902. Correspondence and requests for materials should be addressed to M.I.Y (email: mohammad.younis@kaust.edu.sa)

a. Internal resonance 2:1

To study the internal resonance among two vibration modes of amplitude u_1 and u_2, the below coupled ordinary differential equations can be used[1]

$$\begin{aligned} &\ddot{u}_1 + \omega_1^2 u_1 + 2\mu_1 \dot{u}_1 = \alpha_1 u_1 u_2 \\ &\ddot{u}_2 + \omega_2^2 u_2 + 2\mu_2 \dot{u}_2 = \alpha_2 {u_2}^2 + F\cos(\Omega t) \end{aligned} \tag{S.1}$$

where α_1, α_2, μ_1, μ_2, Ω and F are nondimensional constants defined in Table S.1.

Table S.1: The nondimensional constants

Parameters	Definition	Values
ω_1	1st natural frequency	1
ω_2	3rd natural frequency	2
α_1	Coupling coefficient	1
α_2	Coupling coefficient	1
μ_1	Damping coefficient	1
μ_2	Damping coefficient	2
F	Forcing Amplitude	200

We seek a first-order uniform expansion using the multiple scale method:

$$\begin{aligned} &u_1(t,\varepsilon) = u_{10}(T_0,T_1) + \varepsilon u_{11}(T_0,T_1) + ... \\ &u_2(t,\varepsilon) = u_{20}(T_0,T_1) + \varepsilon u_{21}(T_0,T_1) + ... \end{aligned} \tag{S.2}$$

where ε is a small nondimensional bookkeeping parameter. T_0 and T_1 represent the two-time scales presented as

$$T_0 = \text{t and } T_1 = \varepsilon t \tag{S.3}$$

The temporal operators can be expended as follows:

$$\frac{\partial}{\partial t} = D_0 + \varepsilon D_1 + ... \text{and } \frac{\partial^2}{\partial t^2} = {D_0}^2 + 2\varepsilon D_0 D_1 + ... \tag{S.4}$$

where $D_n = \dfrac{\partial}{\partial T_n}$.

Then (S.1) can be written as

$$\begin{aligned} &\ddot{u}_1 + \omega_1^2 u_1 + 2\varepsilon\mu_1 \dot{u}_1 = \varepsilon\alpha_1 u_1 u_2 \\ &\ddot{u}_2 + \omega_2^2 u_2 + 2\varepsilon\mu_2 \dot{u}_2 = \varepsilon\alpha_2 {u_2}^2 + \varepsilon F\cos(\Omega t) \end{aligned} \tag{S.5}$$

To analyze the 2:1 internal resonance, we let

$$\omega_2 = 2\omega_1 + \varepsilon\sigma_1 \text{ and } \Omega = \omega_2 + \varepsilon\sigma_2 \tag{S.6}$$

where σ_1 and σ_2 represent the detuning parameters. σ_1 represents the nearness of the second resonance frequency to the double of first one and σ_2 represents the nearness of the excitation frequency to the second resonance frequency. In our case, they represent the effect of the electrothermal voltage V_{Th} on the 1st and 3rd resonance frequencies.

Solving these equations using the multiple-scale method, the general solutions of u_1 and u_2 are given by[1]:

$$\begin{aligned} u_1(T_0,T_1) &= A_1(T_1)e^{i\omega_1 T_0} + cc \\ u_2(T_0,T_1) &= A_2(T_1)e^{i\omega_2 T_0} + cc \end{aligned} \tag{S.7}$$

Expressing the complex-value of the amplitude into polar forms, u_1 and u_2 can be written as

$$\begin{aligned} u_1(T_0,T_1) &= \frac{1}{2}a_1(T_1)e^{i(\omega_1 T_0+\beta_1(T_1))} + cc \\ u_2(T_0,T_1) &= \frac{1}{2}a_2(T_1)e^{i(\omega_2 T_0+\beta_2(T_1))} + cc \end{aligned} \tag{S.8}$$

where a_1, a_2, β_1 and β_2 are the modulation parameters representing the amplitude and phase of the resulting motion of mode 1 and 2, respectively.
For the steady state response, two possibilities are found[1]. The first possibility, that represents essentially the solution of the linear problem, is given by

$$a_1 = 0 \text{ and } a_2 = \frac{F}{2\omega_1\sqrt{\mu_2^2 + \sigma_2^2}} \tag{S.9}$$

For the second possibility, a_2 is given by

$$a_2 = \frac{4\omega_1}{|\alpha_1|}\sqrt{\mu_1^2 + \frac{1}{4}(\sigma_1 + \sigma_2)^2} \tag{S.10}$$

We note here that the amplitude of the only mode excited is independent from the amplitude of the excitation.

a_1 is given by solving the 4th order equation

$$\frac{\alpha_2^2 a_1^4}{16} + a_1^2\left[\frac{4\mu_2\mu_1\omega_2\omega_1}{2\alpha_1} + \frac{2\sigma_2\omega_2\omega_1(\sigma_1+\sigma_2)}{\alpha_1}\right] + \omega_2^2 a_2^2\left(\mu_2^2 + \sigma_2^2\right) - \frac{F^2}{4} = 0 \tag{S.11}$$

Unlike the amplitude of the second-mode, the amplitude of the first-mode is dependent on the amplitude of the excitation of the second-mode.

In order to illustrate the possible character of the response, we plot the variation of the amplitude a_1 and a_2 as a function of the amplitude of the excitation F by using the parameter in Table S.1 and by assuming that the detuning parameters are zeros, Figure S.1.

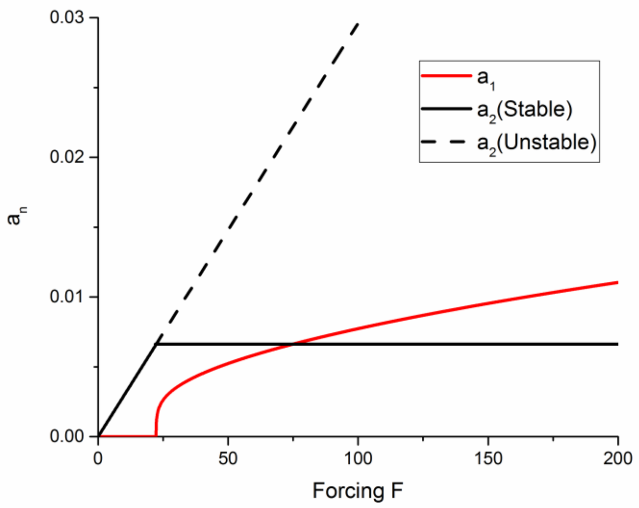

Figure S.1: Amplitude of the response as a function of the amplitude of excitation when $\sigma_1 = \sigma_2 = 0$.

Figure S.2 shows frequency responses as varying the detuning parameters σ_1, which represents the effect of the electrothermal voltage V_{Th} in our case.

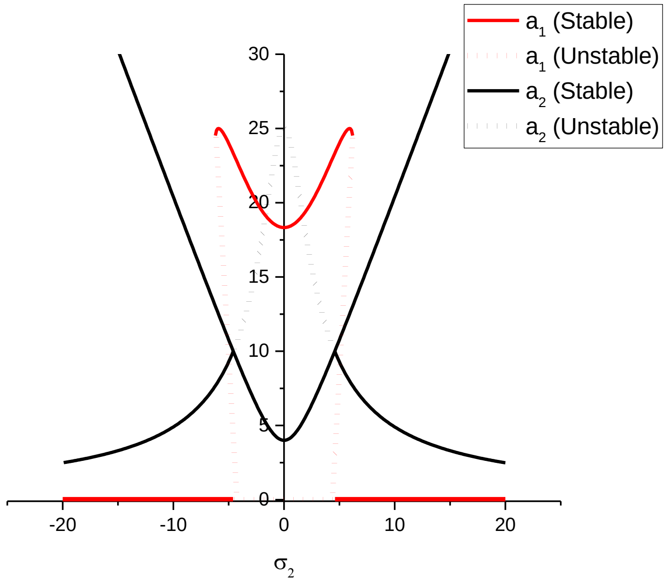

(a)

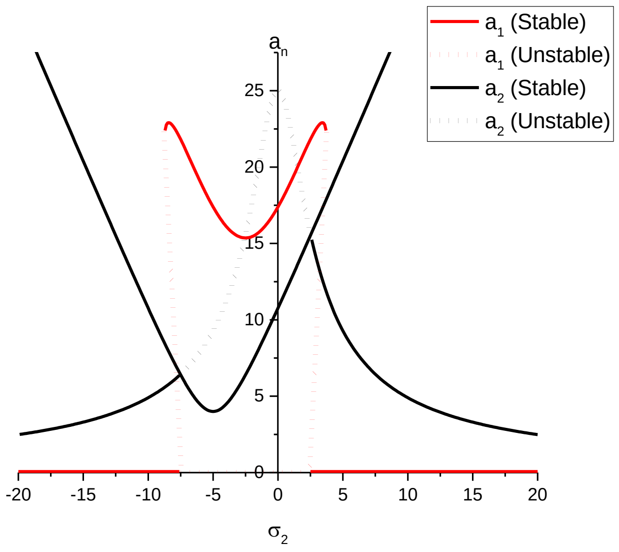

(b)

Figure S.2: Frequency-response curves around the second natural frequency. (a) $\sigma_1 = 0$. (b) $\sigma_1 = 5$.

b. Veering using an Arch under Electrothermal and Electrostatic Actuation

Here we present modeling and simulation results for an arch under electrothermal and electrostatic actuation. The device under consideration consists of an in-plane clamped-clamped shallow arch beam, Figure S.3, of initial shape $\hat{w}_0\,(x)$ governed by

$$\hat{w}_0 = \frac{1}{2}\hat{b}_0\left(1-\cos(\frac{2\pi x}{l})\right) \qquad \text{(S.12)}$$

where $\hat{b}_0$ represents the rise at the mid-point of the arch. The arch is actuated electrothermally by a DC voltage V_{Th} and electrostatically by a DC polarization voltage V_{DC} and an AC harmonic voltage of amplitude V_{AC} and of frequency $\hat{\Omega}$, and is subjected to viscous damping of coefficient ĉ. It has a Young's modulus *E* and a material density ρ. It is of length *l*, width *b*, and thickness *h*. It is assumed to have a rectangular cross section area $A = bh$ and a moment of inertia $I = bh^3/12$. The arch is separated from a stationary electrode with a gap width *d* and with a dielectric constant of the medium *ε*. The electrothermal voltage V_{Th} is applied between the anchors of the arch inducing a current I_{Th} passing through the arch beam that heat up it and controls it's internally induced axial stress.

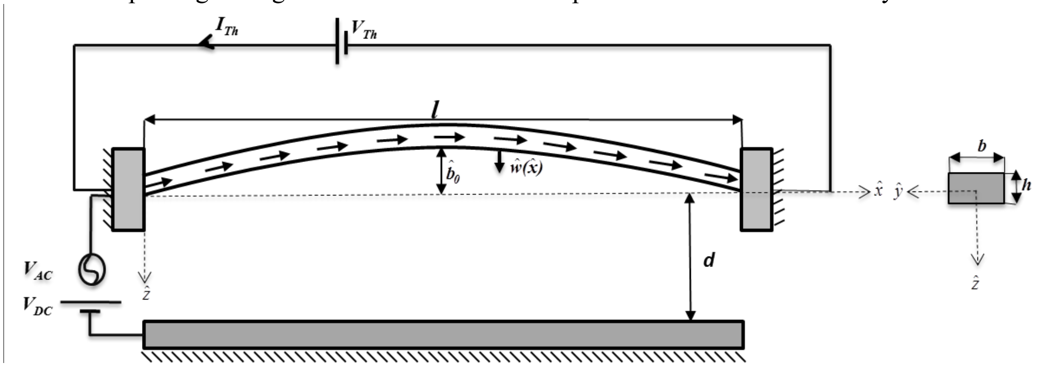

Figure S.3: Schematic of an electrothermally and electrostatically actuated clamped-clamped shallow arch.

The Fourier's law equation governs the thermal distribution inside the beam due V_{Th}, which can be written as

$$-k\frac{d^2T}{d\hat{x}^2} = \frac{\sigma_e {V_{Th}}^2}{l^2} \qquad \text{(S.13)}$$

Solving (S.13), assuming that the temperature at the ends of the microbeam is equal to the ambient temperature T_a, gives a close form solution of the distribution of the temperature:

$$T[\hat{x}] = \frac{\sigma_e {V_{Th}}^2}{2k}\left(\frac{\hat{x}}{l} - \frac{\hat{x}^2}{l^2}\right) + T_a \qquad \text{(S.14)}$$

The variation of the temperature along the microbeam induces a compressive stress expressed as

$$\hat{S}_{Th} = \alpha EA\int_0^l \left(T[\hat{x}] - T_a\right)d\hat{x} \qquad \text{(S.15)}$$

where α is the coefficient of thermal expansion, which is assumed to be independent of temperature in this study.

The governing equation of motion of the shallow arch under consideration, Figure S2.1, describing its transverse deflection $\hat{w}(\hat{x},\hat{t})$ is written as follows:

$$\rho bh\frac{\partial^2 \hat{w}}{\partial \hat{t}^2} + \hat{c}\frac{\partial \hat{w}}{\partial \hat{t}} + EI\frac{\partial^4 \hat{w}}{\partial \hat{x}^4} + \left(\frac{\partial^2 \hat{w}}{\partial \hat{x}^2} + \frac{d^2 \hat{w}_0}{d\hat{x}^2}\right)\left[\hat{N} - \frac{EA}{2l}\int_0^l \left(\left(\frac{\partial \hat{w}}{\partial \hat{x}}\right)^2 + 2\frac{\partial \hat{w}}{\partial \hat{x}}\frac{d\hat{w}_0}{d\hat{x}}\right)d\hat{x}\right] = \frac{1}{2}\varepsilon b\frac{(V_{DC} + V_{AC}\cos(\hat{\Omega}t))^2}{\left(d - \hat{w} - \hat{w}_0\right)^2} \qquad \text{(S.16)}$$

The microbeam is subjected to the following boundary conditions:

$$\hat{w}(0,\hat{t}) = \hat{w}(l,\hat{t}) = 0 \text{ and } \left.\frac{d\hat{w}}{d\hat{x}}\right|_{(0,\hat{t})} = \left.\frac{d\hat{w}}{d\hat{x}}\right|_{(l,\hat{t})} = 0 \qquad \text{(S.17)}$$

where $\hat{x}$ is the position along the microbeam and $\hat{t}$ is time. The term $\hat{N} = \hat{S}_{Th} - \hat{N}_0$ represents the axial load due to the residual axial load, where $\hat{N}_0$ arising from the fabrication process and the compressive axial load and $\hat{S}_{Th}$ govern by (S.15).

For convenience, we introduce the nondimensional variables as below:

$$w = \frac{\hat{w}}{d};\ x = \frac{\hat{x}}{l}\ ;\ t = \frac{\hat{t}}{T};\ w_0 = \frac{\hat{w}_0}{d} = \frac{1}{2}b_0\left(1 - \cos(2\pi x)\right) \text{ and } b_0 = \frac{\hat{b}_0}{d} \qquad \text{(S.18)}$$

where $T = \sqrt{\frac{\rho bhl^4}{EI}}$ is a time scale. Substituting (S.18) into (S.16) and (S.17), we obtain the nondimensional equation of motion of the beam

$$\frac{\partial^2 w}{\partial t^2} + c\frac{\partial w}{\partial t} + \frac{\partial^4 w}{\partial x^4} + \left(\frac{\partial^2 w}{\partial x^2} + \frac{d^2 w_0}{dx^2}\right)\left[N - \alpha_1\int_0^1 \left[\left(\frac{\partial w}{\partial x}\right)^2 + 2\frac{\partial w}{\partial x}\frac{dw_0}{dx}\right]dx\right] = \alpha_2\frac{(V_{DC} + V_{AC}\cos(\Omega t))^2}{\left(1 - w - w_0\right)^2} \qquad \text{(S.19)}$$

Subjected to the nondimensional boundary conditions

$$w(0,t) = w(1,t) = 0 \text{ and } \left.\frac{dw}{dx}\right|_{(0,t)} = \left.\frac{dw}{dx}\right|_{(1,t)} = 0 \qquad \text{(S.20)}$$

The nondimensional parameters appearing in (S.20) are defined as below:

$$\alpha_1 = 6\left(\frac{d}{h}\right)^2\ ;\ \alpha_2 = \frac{6\varepsilon l^4}{Eh^3 d^3};\ c = \frac{l^2}{EI}\hat{c}\ ;\ N = N_0 + S_{Th}\ ;\ N_0 = \frac{l^2}{EI}\hat{N}_0\ ;\ S_{Th} = \frac{l^2}{EI}\hat{S}_{Th}; \qquad \text{(S.21)}$$

Next, we split the deflection of the arch into a static deflection induced by the electrothermal voltage $w_{th}(x)$ and a deflection induced by the electrostatic force $w_{ef}(x,t)$:

$$w(x,t) = w_{th}(x) + w_{ef}(x,t) \qquad \text{(S.22)}$$

where the equation governing the static deflection due to the electrothermal voltage is written as

$$\frac{d^4 w_{th}}{dx^4}+\left(\frac{d^2 w_{th}}{dx^2}+\frac{d^2 w_0}{dx^2}\right)\left[N-\alpha_1\int_0^1\left[\left(\frac{dw_{th}}{dx}\right)^2+2\frac{dw_{th}}{dx}\frac{dw_0}{dx}\right]dx\right]=0 \qquad \text{(S.23)}$$

Subjected to the nondimensional boundary conditions

$$w_{th}(0)=w_{th}(1)=0 \text{ and } \left.\frac{dw_{th}}{dx}\right|_{x=0}=\left.\frac{dw_{th}}{dx}\right|_{x=0}=0 \qquad \text{(S.24)}$$

Following[3], (S.22) can be solved exactly and by assuming it to be of the form

$$w_{th}(x)=\frac{1}{2}q(1-\cos(2\pi x)) \qquad \text{(S.25)}$$

where q is given by:

$$q=a_{th}-b_0 \qquad \text{(S.26)}$$

where a_{th} is the solution of the following characteristic equation:

$$a_{th}{}^3-\left(\frac{2}{\alpha_1\pi^2}N+b_0{}^2-\frac{8}{\alpha_1}\right)a_{th}-\frac{8}{\alpha_1}b_0=0 \qquad \text{(S.27)}$$

The governing equation for the dynamic deflection due to the electrostatic load $w_{ef}(x,t)$ can be obtained by assuming $\Psi(x)=w_{th}(x)+w_0(x)$ in (S2.8) and dropping the terms representing the equilibrium position (S.23), which yield

$$\frac{\partial^2 w_{ef}}{\partial t^2}+c\frac{\partial w_{ef}}{\partial t}+\frac{\partial^4 w_{ef}}{\partial x^4}+\frac{\partial^2 w_{ef}}{\partial x^2}\left[N-\alpha_1\int_0^1\left[\left(\frac{\partial w_{ef}}{\partial x}\right)^2+2\frac{\partial w_{ef}}{\partial x}\frac{d\Psi}{dx}\right]dx\right]$$
$$-\alpha_1\frac{\partial^2 w_{ef}}{\partial x^2}\int_0^1\left[\left(\frac{d\Psi}{dx}\right)^2-\left(\frac{dw_0}{dx}\right)^2\right]dx-\alpha_1\frac{d^2\Psi}{dx^2}\int_0^1\left[\left(\frac{\partial w_{ef}}{\partial x}\right)^2+2\frac{\partial w_{ef}}{\partial x}\frac{d\Psi}{dx}\right]dx=\alpha_2\frac{(V_{DC}+V_{AC}\cos(\Omega t))^2}{\left(1-w_{ef}-\Psi\right)^2} \qquad \text{(S.28)}$$

To solve (S.28), we refer to the Galerkin discretization to yield a reduced order model. Therefore the deflection induced by the electrostatic force is expressed as

$$w_{ef}(x,t)=\sum_{i=0}^{n}u_i(t)\varphi_i(x) \qquad \text{(S.29)}$$

where $u_i(t)$ (i=0, 1, 2...n) denotes the nondimensional modal coordinates and $\varphi_i(x)$ (i=0, 1, 2...n) denotes the undamped mode shapes of the shallow arch under electrothermal, axial, load governed by[1]

$$\varphi^{iv}+\varsigma\varphi''-4\alpha_1 a_{th}{}^2\pi^3\cos(2\pi x)\int_0^1\varphi'\sin(2\pi x)dx-\omega^2\varphi=0 \qquad \text{(S.30)}$$

where ω is the natural frequency and $\varsigma=4\pi^2(1-\frac{b_0}{a_{th}})$.

The general solution of (S.30) is given by

$$\varphi(x)=c_1\cos(s_1x)+c_2\sin(s_1x)+c_3\cosh(s_2x)+c_4\sinh(s_2x)+c_5\cos(2\pi x) \qquad \text{(S.31)}$$

with the associated boundary condition:

$$\varphi(0)=\varphi(1)=0 \text{ and } \left.\frac{d\varphi}{dx}\right|_{x=0}=\left.\frac{d\varphi}{dx}\right|_{x=1}=0 \qquad \text{(S.32)}$$

where $s_{1,2}=\sqrt{\pm\frac{1}{2}\varsigma+\sqrt{\frac{1}{4}\varsigma^2+\omega^2}}$.

The constants c_i are determined by substituting (S.31) into the boundary conditions (S.31), and (S.32) into (S.30). This yields five algebraic equations for the coefficients c_i and the natural frequency ω, which are solved for a fixed electrothermal voltage. The variation of the first and third resonance frequencies of the arch as varying the electrothermal voltage V_{Th} is shown in Figure S2.2. The first natural frequency increases as we increase the compressive load induced by V_{Th}. The third natural frequency decreases as increasing V_{Th} and then starts to increase when the first natural frequency starts to flatten due to the veering phenomenon.

Next, we proceed to determine the forced-vibration response of the arch when subjected to electrostatic loading, in addition to the electrothermal load. Toward this, we multiply (S.28) by $(1-w_{ef}-\Psi)^2$ and substitute (S.23) into (S.28), multiply the outcome by the mode shape φ_j and integrate over the beam domain (from 0 to 1), which yields n differential equations in terms of the modal coordinates $u_i\ (t)$, which can be solved numerically using time integration algorithms.

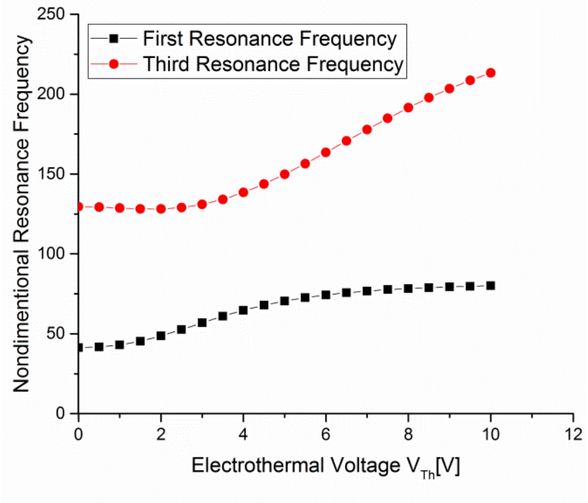

Figure S.4: Variation of the first and third resonance frequencies under the electrothermal voltage.

Figures S.5a and S.5b show the simulation results of two frequency sweep responses under electrostatic force before and after the veering regime, respectively, similar to Figure 9 in the main manuscript. Before veering, amplitude of vibration of the first resonance frequency is shown to be higher than that of the third resonance frequency, Figure S.5a. After veering, Figure S.5b, totally the opposite behavior is observed.

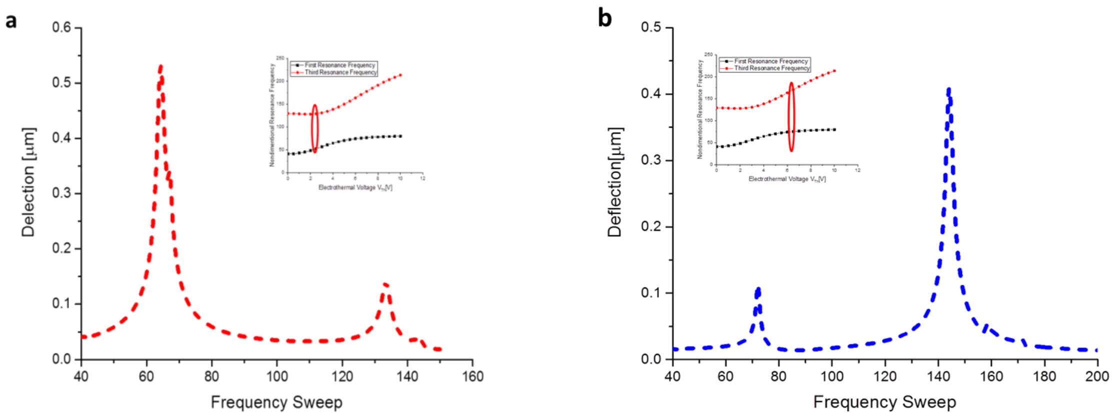

Figure S.5: Simulation results for the frequency sweep response of the arch using electrostatic excitation in the neighborhood of the first and the third resonance frequencies. (a) Before veering, V_{Th}=2.5V, the frequency is swept for V_{DC} = 30V and V_{AC} = 40V. (b) After veering, V_{Th} = 6.5V, the frequency is swept for V_{DC} = 50V and V_{AC} = 50V. The vibrational amplitude of the 3rd resonance after veering is amplified more than that of the 1st resonance frequency.

References:

1. Nayfeh, A. H. & Mook, D. T. *Nonlinear Oscillations* (Wiley Classics Library Edition. John Wiley & Sons, 1995).
2. Nayfeh A. H. *Nonlinear Interactions* (Wiley Classics Library Edition. John Wiley & Sons, 2000).
3. A. H. Nayfeh, W. Kreider, and T. Anderson, "Investigation of natural frequencies and mode shapes of

buckled beams," *AIAA journal*, vol. 33, pp. 1121-1126, 1995.
